# Associations of Inherited Chromosomally‐Integrated Human Herpesvirus 6 With Dementia Incidence, Inflammation, and Other Dementia Risk Factors in the UK Biobank

**DOI:** 10.1002/jmv.71085

**Published:** 2026-07-30

**Authors:** Zhong Qiya, Marko Mandic, Tim Waterboer, Xie Ruijie, Hermann Brenner, Ben Schöttker

**Affiliations:** ^1^ Division of Clinical Epidemiology of Early Cancer Detection German Cancer Research Center (DKFZ) Heidelberg Germany; ^2^ Medical Faculty, Heidelberg University Heidelberg Germany; ^3^ Division of Infections and Cancer Epidemiology German Cancer Research Center (DKFZ) Heidelberg Germany; ^4^ Cancer Prevention Graduate School German Cancer Research Center (DKFZ) Heidelberg Germany; ^5^ Network Aging Research (NAR) Heidelberg University Heidelberg Germany

**Keywords:** Alzheimer's disease, cohort study, dementia, inherited chromosomally‐integrated human herpesvirus 6, vascular dementia

## Abstract

The infection theory of dementia states that viral infections and chronic inflammation play a role in its pathogenesis. We aimed to test whether testing positive for inherited chromosomally‐integrated human herpesvirus 6 (iciHHV‐6) is associated with an increased dementia incidence, inflammation, and other dementia risk factors. We included *n* = 247,731 participants of the UK Biobank in the analysis, of whom *n* = 3388 (1.4%) tested positive for iciHHV‐6. Linear and logistic regression models were performed to assess the associations between iciHHV‐6 with HHV‐6 antigens, blood‐based biomarkers of inflammation, and other dementia risk factors. Cox proportional hazards regression models were applied to assess the associations of iciHHV‐6 with all‐cause dementia, Alzheimer's disease (AD), and vascular dementia (VD). Subjects with iciHHV‐6 exhibited statistically significantly higher antibody responses to the HHV‐6 antigens IE1A (*p* = 0.002) and IE1B (*p* < 0.001). IciHHV‐6 positivity was significantly more frequent among subjects with European or Chinese ethnicity, with lower education, higher alcohol consumption, current smoking, and longer telomere length. Interestingly, iciHHV‐6 positive subjects had lower C‐reactive protein (CRP) levels (*p* = 0.029). All other inflammatory biomarkers did not differ according to iciHHV‐6 status. Overall, 6615 participants were diagnosed with all‐cause dementia during a median of 13.6 years, including 3340 with AD and 1708 with VD. There was no significant association between iciHHV‐6 positivity and the risk of any dementia outcome. IciHHV‐6 positivity was not a risk factor for dementia outcomes or increased inflammation in this large study, but was associated with higher antibody responses against HHV‐6 antigens, ethnicity, telomere length, and lifestyle factors.

**Abbreviations**
AD: Alzheimer's disease
APOE: apolipoprotein E
Aβ: amyloid beta
BMI: body mass index
CI: confidence interval
HHV‐6: human herpesviruses 6
HR: hazard ratio
iciHHV‐6: inherited chromosomally‐integrated human herpesvirus 6
RCT: randomized controlled trial
SD: standard deviation
VD: vascular dementia

## 1 Introduction

Dementia is a neurological syndrome that induces cognitive, behavioral, and psychological impairment among the elderly [1]. To date, approximately 50 million people are suffering from dementia globally [2]. Alzheimer's disease (AD) accounts for 60‐70% of dementia cases, followed by vascular dementia (VD) with 20% [3].

Recently, an increasing number of studies have implicated viruses, especially human herpesviruses, as biological risk factors of dementia [4]. The human herpesviruses (HHV) 1, 2, 6, 7, and the Epstein‐Barr virus (EBV), also known as HHV‐4, were identified as risk factors for AD or all‐cause dementia [5, 6, 7, 8]. For instance, Carbone et al. reported significantly higher HHV‐6 positivity from peripheral blood leukocytes in AD patients than in healthy controls [9]. The underlying mechanism might be related to neuroinflammation, the production of Amyloid beta (Aβ), and its pathological deposition in the brain [7]. Besides being considered a risk factor for AD, HHV‐6 infection may also play a role in multiple other neurological diseases, including seizures and encephalitis [7, 8].

HHV‐6 is distinguished from other human herpesviruses by its ability to integrate its DNA into human chromosomes, which can be passed on to the next generation [10]. The inherited chromosomally integrated HHV‐6 (iciHHV‐6) is horizontally transmitted from a parent with HHV‐6 to a child, and exists in every somatic cell [11, 12]. The prevalence of iciHHV‐6 in the general population is only around 0.5%–1%, with differences according to ethnicity [13]. For example, the prevalence observed in the United Kingdom (UK) (0.8%‐1.5%) [14] is higher than that in Japan (0.2%–0.6%) [15] and Canada (0.6%) [16].

It has been suggested that iciHHV‐6 may co‐localize with Alzheimer's disease risk loci, potentially influencing amyloid plaque formation and accelerating the progression of tau neurofibrillary tangles (NFTs) [17]. Furthermore, a large cross‐sectional study from Canada observed that a positive iciHHV‐6 status is associated with angina pectoris [16], a risk factor for vascular dementia (VD) [18]. A case study of two patients with positive iciHHV‐6 status and cognitive dysfunction showed improved symptoms after anti‐retroviral therapy [19]. Motivated by these results, we conducted the first study to assess the associations of iciHHV‐6 positivity with all‐cause dementia, AD, and VD incidence. We further explored the associations of iciHHV‐6 status with dementia risk factors and, as neuroinflammation may play a role as the underlying mechanism, we put an emphasis on the associations of iciHHV‐6 with biomarkers of inflammation.

## 2 Methods

### 2.1 Data Source

This study is based on the UK Biobank, which recruited more than half a million participants who attended one of the 22 study assessment centers distributed across England, Scotland, and Wales between March 2006 and October 2010 [20]. The age range at baseline was 40–69 years with very few outliers. Data were obtained via questionnaires, interviews, health records, physical measures, and biological samples, including blood, urine, and saliva. All participants voluntarily provided signed informed consent. Longitudinal data on health‐related outcomes were mainly acquired by linkage to routinely available data from the UK National Health Service, death registrations, cancer registries, hospital records, and primary care data.

### 2.2 Assessment of iciHHV‐6 and HHV‐6 Serostatus

TaqMan qPCR was used to determine the iciHHV‐6 status by screening DNA samples of all UK Biobank participants. After excluding missing samples, controls, replicates, and withdrawn samples, the iciHHV‐6 status could be determined for 416,347 study participants. The methods are described in detail elsewhere [21]. In brief, the HHV‐6 genome comprises a long unique (U) region spanning U2 to U100, flanked by left and right Direct Repeat (DR) regions (DR1 and DR6). Additionally, telomere‐like regions are present at the ends of the genome. Primers and probes targeting the U7 and DR1 regions were used for the molecular assays. Both assays amplify from iciHHV‐6A and iciHHV‐6B. In addition, a β‐globin assay was used to target a human single‐copy reference. The TaqMan qPCR assay cycle threshold (Ct) values for the U7, DR and β‐globin genes were used to automatically derive the screen results. Based on these results for the two regions and the β‐globin reference, the measurements were classified as follows:

- ΔU7 Ct = β‐globin Ct – U7 Ct
- ΔDR1 Ct = β‐globin Ct – DR1 Ct
- If ΔU7 Ct < 6 and ΔDR1 Ct < 6, sample scored as “Likely iciHHV‐6 positive” (*n* = 5635).
- If ΔU7 Ct ≥ 6 and ΔDR1 Ct < 6, sample scored as “DR‐only positive” (*n* = 252).
- If ΔU7 Ct < 6 and ΔDR1 Ct ≥ 6, sample scored as “U‐only positive” (*n* = 1).
- If 6 ≤ ΔU7 Ct or 6 ≤ ΔDR1 Ct < 8, sample scored as “Unlikely iciHHV‐6 positive” (*n* = 154).
- If ΔU7 Ct ≥ 8 and ΔDR1 Ct ≥ 8, sample scored as “iciHHV‐6 negative” (*n* = 410,458).

In certain instances, manual assignment of the status was preferred when it appeared to be more appropriate. Only subjects classified as “likely iciHHV‐6 positive” were considered as “iciHHV‐6 positive” in our study, and subjects with “unlikely iciHHV‐6 positive” or “negative” classification were considered as “iciHHV‐6 negative.” If only the DR region or U region reached scores above positivity thresholds, these were inconclusive results, and we excluded them from the data set.

Almost 10,000 UK Biobank participants were randomly selected to test the serostatus of various viruses in the lab of the division of Infections and Cancer Epidemiology at the German Cancer Research Center (DKFZ), of which 9695 samples passed validity checks and were available for data analysis. The entire multiplex serology was conducted over 2 weeks, and the methods are described in detail elsewhere [22]. HHV‐6 seropositivity was defined as positive if one or more of the following three antigens were above the cut‐off value of 100 mean fluorescence intensity (MFI) units: IE1A (Immediate‐early 1 protein of HHV‐6A), IE1B (Immediate‐early 1 protein of HHV‐6B), or HHV‐6 virion protein p101k (late antigen).

### 2.3 Biomarkers of Inflammation

Serum C‐reactive protein (CRP) levels (mg/L) were measured by Beckman Coulter AU5800 immunoturbidimetric high‐sensitivity assay, and serum albumin levels were determined through bromocresol green (BCG) analysis using the same instrument [23]. Peripheral blood samples from UK Biobank participants were tested within 24 h of blood draw at the UK Biobank central laboratory using a Beckman Coulter LH750 Hematology Analyzer. Neutrophil, lymphocyte, and monocyte counts were calculated by the instrument from differential blood cell counts (operating range 0.00–900.00 × 109 cells/L), whereas platelet counts were assessed directly as instrument measurements (operating range 0.00–5000 × 109 cells/L) [24]. The systemic immune‐inflammation index (SII), neutrophil‐to‐lymphocyte ratio (NLR), platelet‐to‐lymphocyte ratio (PLR), lymphocyte‐to‐monocyte ratio (LMR), and prognostic nutritional index (PNI) were calculated as follows: SII = (neutrophil count × platelet count)/lymphocyte count, NLR = neutrophil count/lymphocyte count, PLR = platelet count/lymphocyte count, LMR = lymphocyte count/monocyte count, and PNI = serum albumin level+ 0.005 × 1000 × lymphocyte count [25, 26].

In the UK Biobank, proteomic profiling was conducted on EDTA plasma samples collected at baseline using the Olink Explore 3072 platform (Olink Proteomics, Uppsala, Sweden), with a Proximity Extension Assay (PEA) in a subset of *n* = 54,219 UK Biobank participants. The platform detects 2923 unique proteins. The assay protocols, including sample handling and selection procedures, have been described previously [27]. Our group previously identified six proteins from the OLINK Target 96 inflammation panel (which is a part of the OLINK Explore 3072 platform), which were strongly associated with incident all‐cause, AD, or VD dementia in a German cohort study and were not strongly correlated with each other [28]. These six proteins are IL‐6 (interleukin 6), TNF (tumor necrosis factor), CX3CL1 (fractalkine), EN‐RAGE (extracellular newly identified receptor for advanced glycation end products binding protein), LAP TGF‐β1 (latency‐associated peptide‐transforming growth factor‐β−1), and VEGF‐A (vascular endothelial growth factor‐A). We focused on these six inflammation‐related proteins in the analyzes.

### 2.4 Assessment of Conventional Dementia Risk Factors

Conventional dementia risk factors were selected as covariates, which contained sociodemographic factors (age, sex, ethnicity, education, and household income), genetic data (APOE ε4 allele status, and telomere length), life‐style factors (physical activity, alcohol consumption, smoking status, and BMI), comorbidity (history of depression, anxiety, coronary heart disease, history of stroke, hypertension, diabetes, and arthritis), and physical function (grip strength, and hearing impairment). Adjustment for APOE ε4 is important in this context because it is the strongest individual risk factor for AD after age. Carrying one ε4 allele is associated with an about threefold increased AD risk, and carrying two alleles with an about 15‐fold increased risk [29]. Details of the assessment methods for the covariates are outlined in Supplementary Text S1.

### 2.5 Ascertainment of Incident Dementia Outcomes

We acquired incidence of all‐cause dementia, AD, and VD through hospital admission records and death registries [30]. The time under observation was from the day of the first attendance at the assessment center till a dementia diagnosis, death, or end of follow‐up, which was based on the hospital records availability in the study regions (10/2022 for England, 08/2022 for Scotland, and 05/2022 for Wales). Among the included participants, the median follow‐up time for all‐cause dementia was 13.6 years (minimum: 0.2 years, maximum: 16.5 years).

### 2.6 Statistical Analyzes

#### 2.6.1 Study Population

**Figure 1:**
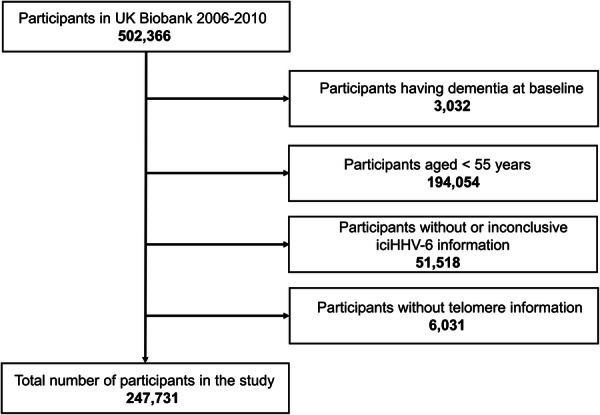
Flow‐chart of the study population.

From the initial 502,366 participants recruited in the UK Biobank, individuals with dementia at baseline (*n* = 3032), those younger than 55 years (*n* = 194,054), participants missing or inconclusive iciHHV‐6 measurements (*n* = 51,518), and those lacking telomere information (*n* = 6031) were excluded (Figure 1). The final analysis included 247,731 participants in this study. Subjects aged 40‐55 years were excluded because the dementia incidence in this age group was very low (0.2%). Telomere length was considered an important confounder because iciHHV‐6 is located towards the end of the chromosomes.

#### 2.6.2 Statistical Software and Significance Level

The R statistical software was utilized to perform all statistical analyzes in the UKB Research Analysis Platform (UKB‐RAP). A two‐sided *p‐*value less than 0.05 was defined as statistical significance for all statistical tests.

#### 2.6.3 Multiple Imputation

As the assumption of missing at random (MAR) was considered reasonable, multiple imputation using the Markov chain Monte Carlo (MCMC) method was performed to generate five imputed datasets. This approach was used to address missing values among covariates included in the most comprehensively adjusted model. The covariate with the highest proportion of missing data was physical activity (26.7%). While there is no universally accepted threshold for an acceptable proportion of missing data, a missingness of up to 50% is often considered acceptable when the MAR assumption holds [31]. PROC MIANALYZE was used for subsequent regression analyzes with the imputed 5 datasets [32].

#### 2.6.4 Cross‐Sectional Associations of iciHHV‐6 With HHV‐6 Seropositivity, HHV‐6 Antigens, Risk Factors of Dementia Outcomes, and Biomarkers of Inflammation

The association of iciHHV‐6 positivity with HHV‐6 seropositivity was assessed with a χ [2] test, and an unadjusted odds ratio (OR) with 95% confidence intervals (95%‐CI) was additionally estimated. Adjusted linear regression was used to assess the association of iciHHV‐6 positivity with the mean fluorescence intensity (MFI) of the antibody responses against the HHV‐6 antigens IE1A, IE1B, and p101k, which were log‐transformed for approximate normal distribution. The same methods were used for the biomarkers of inflammation as dependent variables. Moreover, ORs and 95%CI were assessed for the association of iciHHV‐6 positivity with all traditional dementia risk factors using an adjusted logistic regression model. The same covariates in all adjusted regression models stated above were age, sex, ethnicity, education, household income, APOE ε4 allele status, and telomere length.

#### 2.6.5 Longitudinal Associations of iciHHV‐6 With Incident Dementia Outcomes

We used Cox proportional hazards regression models to explore potential associations of iciHHV‐6 with the dementia outcomes. Covariates as listed in section 2.3 were progressively adjusted in five models: Model 1 adjusted for sociodemographic factors, Model 2 added genetic factors, Model 3 added lifestyle factors, Model 4 added diseases and functional status, and Model 5 added the inflammation‐related biomarker CRP.

## 3 Results

### 3.1 Characteristics of the Study Population

The baseline characteristics of the participants are presented in Table 1. Among the 247,731 participants, 115,628 (46.7%) were male, and 132,103 (53.3%) were female. The majority of 155,235 participants (62.7%) were older than 60 years. The study population was predominantly of European British ethnicity (92.9%). The majority (72.0%) did not carry any APOE ε4 allele, and only 2.2% were positive for APOE ε4/ε4 status. As usual in Western European populations, a substantial proportion of study participants were engaged in an unhealthy lifestyle and were affected by cardiometabolic health conditions. *N* = 34,956 (14.1%) had arthritis. Approx. every 10th participant had a lifetime history of depression (10.4%), whereas current anxiety was rarer (1.3%). Approx. half of the study participants had low grip strength (51.6%), and one third had impaired hearing abilities (30.1%).

**Table 1:** Baseline characteristics and their associations with iciHHV‐6 status.

Risk factors	Total population (*n* = 247,731)	iciHHV‐6 status		
		iciHHV‐6 positive (*n*, %) (*n* = 3,388)	Odds ratio^a^	*p* value^a^
Sociodemographic Factors				
Age (years)				
< 60	92,496 (37.3%)	1297 (1.4%)	Ref.	Ref.
≥ 60	155,235 (62.7%)	2091 (1.3%)	0.95 (0.88; 1.02)	0.167
Sex, *n* (%)				
Female	132,103 (53.3%)	1784 (1.4%)	Ref.	Ref.
Male	115,628 (46.7%)	1604 (1.4%)	1.03 (0.96; 1.11)	0.369
Ethnicity, *n* (%)				
European British	230,103 (92.9%)	3126 (1.4%)	Ref.	Ref.
Asian or Asian British	7040 (2.8%)	56 (0.8%)	0.58 (0.44; 0.76)	**< 0.001**
Black or Black British	936 (0.4%)	6 (0.6%)	0.48 (0.21; 1.06)	0.070
Chinese	9176 (3.7%)	199 (2.2%)	1.61 (1.40; 1.87)	**<** 0.001
Mixed or other	476 (0.2%)	1 (0.2%)	0.15 (0.02; 1.09)	0.061
Education (school years), *n* (%)				
< 10	74,070 (29.9%)	1095 (1.5%)	Ref.	Ref.
≥ 10	173,661 (70.1%)	2293 (1.3%)	0.88 (0.82; 0.95)	0.002
Annual household income (£), *n* (%)				
< 18,000	75,681 (30.6%)	1059 (1.4%)	Ref.	Ref.
18,000– < 51,999	130,400 (52.6%)	1761 (1.4%)	0.99 (0.91; 1.09)	0.989
52,000	41,650 (16.8%)	568 (1.4%)	1.01 (0.90; 1.13)	0.915
Genetic Factors				
APOE ε4 allele status, *n* (%)				
No carriers	178,289 (72.0%)	2423 (1.4%)	Ref.	Ref.
ε2/ε4	5714 (2.3%)	67 (1.2%)	0.87 (0.68; 1.11)	0.266
ε3/ε4	58,366 (23.5%)	815 (1.4%)	0.99 (0.90; 1.10)	0.945
ε4/ε4	5362 (2.2%)	83 (1.5%)	1.09 (0.87; 1.37)	0.460
Telomere length (log Z adjusted)				
< −0.41	95,131 (38.4%)	1261 (1.3%)	Ref.	Ref.
−0.41– < 0.41	82,859 (33.4%)	1115 (1.3%)	1.02 (0.94; 1.10)	0.661
≥ 0.41	69,741 (28.2%)	1012 (1.5%)	1.10 (1.01; 1.20)	**0.022**
Lifestyle Factors				
Physical activity (hours/day), *n* (%)				
< 1	129,254 (50.1%)	1745 (1.4%)	Ref.	Ref.
1– < 2	89,665 (38.9%)	1237 (1.4%)	1.03 (0.95; 1.12)	0.426
≥ 2	28,812 (11.0%)	406 (1.4%)	1.04 (0.92; 1.17)	0.520
Alcohol consumption (g/d), *n* (%)				
Abstainer	75,841 (30.6%)	989 (1.3%)	Ref.	Ref.
Women 0– < 20/men 0– < 40	100,012 (40.4%)	1348 (1.3%)	1.05 (0.96; 1.14)	0.266
Women 20– < 40/men 40– < 60	41,919 (16.9%)	606 (1.4%)	1.14 (1.02; 1.26)	**0.016**
Women ≥ 40/men ≥ 60	29,959 (12.1%)	445 (1.5%)	1.17 (1.04; 1.31)	**0.009**
Smoking status, *n* (%)				
Never	127,396 (51.4%)	1680 ((1.3%)	Ref.	Ref.
Former	98,169 (39.6%)	1362 (1.4%)	1.05 (0.97; 1.13)	0.223
Current	22,226 (9.0%)	346 (1.6%)	1.16 (1.03; 1.31)	**0.014**
BMI (kg/m^2^), *n* (%)				
< 20	4803 (1.9%)	63 (1.3%)	Ref.	Ref.
20– < 25	70,951 (28.6%)	945 (1.3%)	0.99 (0.77; 1.28)	0.946
25– < 30	109,417 (44.2%)	1548 (1.4%)	1.04 (0.81; 1.34)	0.754
30– < 35	45,529 (18.4%)	617 (1.4%)	0.98 (0.75; 1.27)	0.884
≥ 35	17,031 (6.9%)	215 (1.3%)	0.92 (0.69; 1.22)	0.557
Comorbidity				
Hypertension, *n* (%)				
No	163,880 (66.2%)	2257 (1.4%)	Ref.	Ref.
Untreated hypertension	19,180 (7.7%)	254 (1.3%)	0.95 (0.84; 1.09)	0.480
Treated hypertension	64,671 (26.1%)	877 (1.4%)	0.98 (0.90; 1.06)	0.535
Diabetes, *n* (%)				
No	232,179 (93.7%)	3193 (1.4%)	Ref.	Ref.
Yes	15,552 (6.3%)	195 (1.3%)	0.90 (0.77; 1.04)	0.142
Coronary heart disease, *n* (%)				
No	231,421 (93.4%)	3160 (1.4%)	Ref.	Ref.
Yes	16,310 (6.6%)	228 (1.4%)	1.01 (0.87; 1.15)	0.951
History of stroke, *n* (%)				
No	243,317 (98.2%)	3326 (1.4%)	Ref.	Ref.
Yes	4414 (1.8%)	62 (1.4%)	1.02 (0.79; 1.31)	0.905
Lifetime history of depression, *n* (%)				
No	221,994 (89.6%)	3034 (1.4%)	Ref.	Ref.
Yes	25,737 (10.4%)	354 (1.4%)	1.01 (0.90; 1.12)	0.923
Anxiety, *n* (%)				
No	244,557 (98.7%)	3336 (1.4%)	Ref.	Ref.
Yes	3174 (1.3%)	52 (1.6%)	1.20 (0.91; 1.58)	0.206
Arthritis, *n* (%)				
No	212,775 (85.9%)	2909 (1.4%)	Ref.	Ref.
Yes	34,956 (14.1%)	479 (1.4%)	0.99 (0.90; 1.10)	0.927
Physical Function				
Grip strength, *n* (%)				
Low	127,898 (51.6%)	1699 (1.3%)	Ref.	Ref.
Average/high	119,833 (48.4%)	1689 (1.4%)	1.07 (0.99; 1.15)	0.062
Hearing impairment, *n* (%)				
No	173,041 (69.9%)	2318 (1.3%)	Ref.	Ref.
Yes	74,690 (30.1%)	1070 (1.4%)	1.07 (0.99; 1.15)	0.080

*Note:* Bold print: Statistically significant (*p* < 0.05).

Abbreviations: APOE, apolipoprotein E; BMI, body mass index; iciHHV‐6, inherited chromosomally‐integrated human herpesvirus 6.

^a^ Logistic regression adjusted for age, sex, ethnicity, education, household income, APOE ε4 allele status and telomere length.

### 3.2 Cross‐Sectional Associations of Dementia Risk Factors With iciHHV‐6 Positivity

Overall, 3388 out of 247,731 included study participants tested positive for iciHHV‐6 (1.4%). Age, sex, income, BMI, APOE ε4 allele status, comorbidity, and physical function were not associated with iciHHV‐6 positivity. However, compared to participants of European British ethnicity, individuals identifying as Asian or Asian British had statistically significantly 42% lower odds of being iciHHV‐6 positive. Black or Black British ethnicity and mixed/other ethnicity were also associated with substantially lower odds for iciHHV‐6 positivity, but these associations narrowly missed statistical significance. In contrast, subjects with Chinese ethnicity had statistically significantly 61% higher odds of being iciHHV‐6 positive than individuals of European British ethnicity. Furthermore, subjects in the top 30% of the population with regard to telomere length had statistically significantly 10% higher odds of being iciHHV‐6 positive. Moreover, iciHHV‐6 positivity was significantly more frequent among subjects with low education, high alcohol consumption, and current smokers, which are factors that often cluster with each other.

In sensitivity analyzes, we checked whether the associations of a positive iciHHV‐6 status with lifestyle and other factors could be explained by the well‐known prevalence difference of iciHHV‐6 positivity by ethnicity. Restricting the same analysis as shown in Table 1 to individuals with European British ethnicity showed that all statistically significant associations of a positive iciHHV‐6 status with baseline characteristics were virtually unchanged (Supporting Information Table S1).

### 3.3 Cross‐Sectional Associations of iciHHV‐6 Status With HHV‐6 Seropositivity Status

The substantially smaller subset of 4766 participants with available HHV‐6 seropositivity status showed that almost all UK Biobank participants (98.7%) had a past or current HHV‐6 infection (Supporting Information Table 2). Subjects with iciHHV‐6 had an almost threefold higher chance of being HHV‐6 seropositive (OR [95%CI]: 2.92 (0.71–12.02)), but the association was not statistically significant (*p* = 0.119). Individuals positive for iciHHV‐6 had significantly higher MFI of the antibody responses against the HHV‐6 antigens IE1A and IE1B, but no statistically significant difference was observed for p101k. (Table 2).

**Table 2:** Association of iciHHV‐6 positivity with the mean fluorescence intensity (MFI) of the antibody responses against the HHV‐6 antigens IE1A, IE1B, and p101k.

HHV‐6 antigen	iciHHV‐6 negative	iciHHV‐6 positive	*p* value^a^
	Mean MFI (IQR)	Mean MFI (IQR)	
IE1A	204 (110; 375)	326 (173; 535)	0.002
IE1B	221 (114; 478)	2039 (399; 4514)	< **0.001**
p101k	19 (6; 71)	19 (4; 61)	0.160

*Note:* Bold print: Statistically significant (*p* < 0.05).

Abbreviations: HHV‐6, human herpesviruses 6; iciHHV‐6, inherited chromosomally‐integrated human herpesvirus 6. IE1A, Immediate‐early 1 protein of HHV‐6A; IE1B, Immediate‐early 1 protein of HHV‐6B; p101k, HHV‐6 virion protein p101k (late antigen); MFI, mean fluorescence intensity.

^a^ Linear regression adjusted for age, sex, ethnicity, education, household income, APOE ε4 allele status and telomere length with HHV‐6 antigens log transformed to ensure approximate normal distribution.

### 3.4 Cross‐Sectional Associations of iciHHV‐6 Status With Biomarkers of Inflammation

The associations between iciHHV‐6 status and biomarkers of inflammation were not statistically significant, with one exception for CRP (Table 3). Ordinal logistic regression using clinical cut‐offs for CRP‐defined inflammation status revealed that individuals positive for iciHHV‐6 had significantly lower odds of subclinical inflammation (OR [95% CI]: 0.90 [0.82–0.98]), but no significant difference in the odds of acute inflammation (OR [95% CI]: 0.97 [0.82–1.15]) (Table 4).

**Table 3:** Association of iciHHV‐6 status with biomarkers of inflammation.

Biomarkers of inflammation	n_total_	iciHHV‐6 status				p value^a^
		iciHHV‐6 negative		iciHHV‐6 positive		
		*n*	Median (IQR)	*n*	Median (IQR)	
CRP, mg/L	247,731	244,343	1.46 (0.76; 2.96)	3,388	1.43 (0.72; 2.78)	**0.029**
PLR, no unit	247,731	244,343	131 (104; 165)	3,388	132 (105; 166)	0.286
LMR, no unit	247,731	244,343	4.08 (3.17; 5.27)	3,388	4.06 (3.16; 5.26)	0.841
SII, no unit	247,731	244,343	525 (390; 711)	3,388	526 (393; 715)	0.802
NLR, no unit	247,731	244,343	2.14 (1.67; 2.79)	3,388	2.16 (1.68; 2.77)	0.572
PNI, no unit	247,731	244,343	48.7 (46.0; 51.6)	3,388	48.7 (46.0; 51.6)	0.941
IL‐6, NPX	25,535	26,181	0.10 (−0.33; 0.66)	354	0.08 (−0.37; 0.62)	0.999
TNF, NPX	26,123	25,785	0.04 (−0.17; 0.28)	338	0.01 (−0.13; 0.24)	0.189
CX3CL1, NPX	26,430	26,077	0.03 (−0.20; 0.27)	353	0.02 (−0.16; 0.31)	0.631
EN‐RAGE, NPX	26,614	26,264	0.01 (−0.30; 0.33)	350	−0.02 (−0.37; 0.30)	0.956
LAP TGF‐beta‐1, NPX	22,491	22191	0.01 (−0.12; 0.14)	300	0.02 (−0.11; 0.15)	0.737
VEGF‐A, NPX	26,857	26,503	0.05 (−0.35; 0.56)	354	0.08 (−0.30; 0.55)	0.622

*Note:* Bold print: Statistically significant (*p* < 0.05).

Abbreviations: CRP, C‐reaction protein; CX3CL1, fractalkine; EN‐RAGE, extracellular newly identified receptor for advanced glycation end products binding protein; IQR, interquartile range; LMR, lymphocyte‐to‐monocyte ratio; NLR, neutrophil‐to‐lymphocyte ratio; PLR, platelet‐to‐lymphocyte ratio; PNI, prognostic nutritional index; SII, systemic immune‐inflammation index; IL‐6, interleukin 6; TNF, tumor necrosis factor; LAP TGF‐beta‐1, latency‐associated peptide‐transforming growth factor‐β−1; VEGF‐A, vascular endothelial growth factor‐A.

^a^ Linear regression model adjusted for age, sex, ethnicity, education years, household income, APOE ε4 allele status and telomere length; all biomarkers were log transformed.

**Table 4:** Association of iciHHV‐6 status with inflammation based on clinical cut‐offs of CRP levels.

Inflammation	iciHHV‐6 status		
	iciHHV‐6 negative (*n*, %) (*n* = 244,343)	iciHHV‐6 positive (*n*, %) (*n* = 3388)	OR [95%CI]^a^
No inflammation (CRP < 3 mg/L)	186,217 (76.2%)	2635 (77.8%)	Ref
Subclinical inflammation (CRP, 3– < 10 mg/L)	47,566 (19.5%)	607 (17.9%)	**0.90 (0.82; 0.98)**
Acute inflammation (CRP ≥ 10 mg/L)	10,560 (4.3%)	146 (4.3%)	0.97 (0.82; 1.15)

*Note:* Bold print: Statistically significant (*p* < 0.05).

Abbreviations: CI, confidence interval; iciHHV‐6, inherited chromosomally‐integrated human herpesvirus 6; OR, odds ratio.

^a^ Result of ordinal logistic regression model adjusted for age, sex, ethnicity, education years, household income, APOE ε4 allele status and telomere length.

### 3.5 Longitudinal Associations of iciHHV‐6 Status With Dementia Outcomes

A total of 6615 individuals were diagnosed with all‐cause dementia during a median of 13.6 years of follow‐up, including *n* = 3340 with Alzheimer's dementia and *n* = 1708 with vascular dementia. All potential associations between iciHHV‐6 and dementia outcomes were not statistically significant in all of the tested models (Table 5).

**Table 5:** Association of iciHHV‐6 with all‐cause dementia, Alzheimer's dementia and vascular dementia.

Models	All‐cause dementia (n_cases_ = 6615)		Alzheimer's dementia (n_cases_ = 3340)		Vascular dementia (n_cases_ = 1708)	
	iciHHV‐6 negative (*n* = 6513)	iciHHV‐6 positive (*n* = 102)	iciHHV‐6 negative (*n* = 3291)	iciHHV‐6 positive (*n* = 49)	iciHHV‐6 negative (*n* = 1683)	iciHHV‐6 positive (*n* = 25)
		**HR (95%CI)**		**HR (95%CI)**		**HR (95%CI)**
*Model Ⅰ*	Ref.	1.10 (0.91; 1.34)	Ref.	1.05 (0.80; 1.40)	Ref.	1.04 (0.70; 1.54)
*Model Ⅱ*	Ref.	1.10 (0.90; 1.34)	Ref.	1.04 (0.79; 1.38)	Ref.	1.04 (0.79; 1.38)
*Model Ⅲ*	Ref.	1.10 (0.90; 1.34)	Ref.	1.10 (0.90; 1.34)	Ref.	1.04 (0.70; 1.54)
*Model Ⅳ*	Ref.	1.08 (0.89; 1.32)	Ref.	1.03 (0.78; 1.37)	Ref.	0.99 (0.67; 1.48)
*Model Ⅴ*	Ref.	1.08 (0.89; 1.32)	Ref.	1.03 (0.78; 1.37)	Ref.	1.01 (0.68; 1.49)

*Note:* Model I, Adjusted for age, sex, ethnicity, education years and household income. Model II, Adjusted for age, sex, ethnicity, education years, household income, APOE ε4 allele status and telomere length. Model Ⅲ, Adjusted for age, sex, ethnicity, education years, household income, APOE ε4 allele status, telomere length, physical activity and alcohol consumption, smoking status. Model Ⅳ, Adjusted for age, sex, ethnicity, education years, household income, APOE ε4 allele status, telomere length, physical activity, alcohol consumption, smoking status, depression, anxiety, coronary heart disease, history of stroke, hypertension, diabetes, arthritis, BMI, grip strength and hearing impairment. Model Ⅴ, Adjusted for age, sex, ethnicity, education years, household income, APOE ε4 allele status, telomere length, physical activity, alcohol consumption, smoking status, depression, anxiety, coronary heart disease, history of stroke, hypertension, diabetes, arthritis, BMI, grip strength, hearing impairment and CRP

Abreviations: BMI, body mass index; CI, confidence interval; CRP, C‐reactive protein; HR, hazard ratio; NLR, neutrophil‐to‐lymphocyte ratio.

## 4 Discussion

### 4.1 Summary of the Findings

In this large cohort of 247,731 older adults, IciHHV‐6 was not a risk factor for dementia outcomes or inflammation. On the contrary, subjects positive for iciHHV‐6 had lower odds for subclinical inflammation determined by CRP levels of 3–9.9 mg/L. Furthermore, they had statistically significantly higher MFI of the antibody responses against the HHV‐6 antigens IE1A and IE1B, and threefold higher odds for a previous HHV‐6 infection. However, the latter association was not statistically significant because very few study participants had no previous HHV‐6 infection (1.3%). Interestingly, among the dementia risk factors, ethnicity, education level, alcohol consumption, current smoking, and the telomere length of the study participants were statistically significantly associated with iciHHV‐6 positivity.

### 4.2 HHV‐6 Infection, iciHHV‐6, and Alzheimer's Disease

HHV‐6 comprises two subgroups, HHV‐6A and HHV‐6B [33, 34]. The infection rate of HHV‐6B is close to 100% among human beings, while the prevalence of HHV‐6A is unknown [35, 36]. Recently, an autopsy study found that patients with AD present a higher rate of HHV‐6 in the brain than age‐matched cases without AD [37]. Furthermore, Carbone et al. reported significantly higher HHV‐6 positivity from peripheral blood leukocytes in AD patients than in healthy controls [9]. Another study identified an inverse linear correlation between HHV‐6 copy number and performance on cognitive tasks related to orientation, attention‐calculation, and language [38]. While these study results are consistent, they are limited to case‐control designs and can be prone to confounding. As a genetic biomarker, the iciHHV‐6 status might be less prone to confounding, and as it is already present at birth, the timely sequence between exposure and AD outcome is clear [13]. This might explain why our study did not confirm the hypothesis that iciHHV‐6 positivity is a risk factor for AD and other dementia subtypes. We can also exclude measurement errors in the iciHHV‐6 assessment as an alternative explanation because we observed plausible associations with the antibody responses to the HHV‐6 antigens IE1A and IE1B, which are both immediate‐early response antigens. Immediate‐early genes can be expressed at low levels and in a sporadic manner, even without full viral reactivation [39, 40]. In contrast, late response antigens (e.g., p101K) require viral DNA replication and the late transcription machinery [41]. No viral gene expression including miRNAs was detected from the HHV‐6A genomes, indicating that the integrated virus is transcriptionally silent [42]. In line with our findings, previous studies have reported elevated HHV‐6 antibody titers in iciHHV‐6 carriers [36], including children without a history of primary HHV‐6 infection [15].

### 4.3 Associations of iciHHV‐6 With Other Dementia Risk Factors

Moreover, the prevalence of iciHHV‐6 positivity in the UK Biobank (1.4%) was comparable to the prevalences observed in previous population‐based studies from the UK, which ranged from 0.8% to 1.5% [14]. Another plausible finding for the determinants of iciHHV‐6 positivity was the higher frequency among subjects with European or Chinese ethnicity than in other ethnicities (Other Asian, Black, or Mixed) because other studies have also shown regional or ethnic heterogeneity in the iciHHV‐6 positivity prevalence [13, 43]. The association of longer telomere length and iciHHV‐6 positivity might be explained by the fact that the genetic information of HHV‐6A and −6B gets integrated into human DNA, specifically into telomeres towards the end of each chromosome [21]. Thus, the shorter the telomeres, it may be the less likely to detect iciHHV‐6.

As iciHHV‐6 has rarely been assessed in large studies before, we are not aware of other studies showing that it is associated with lower education, higher alcohol consumption, and current smoking. These associations point in the direction of a less healthy lifestyle of subjects with iciHHV‐6 positivity. We can only speculate about the reasons. Maybe the genetic information for HHV‐6 infection and low health‐conscious behavior gets passed on more frequently together.

### 4.4 Inflammation as a Potential Mediator in the Relationship of iciHHV‐6 and AD

As neuroinflammation may play a role as the underlying mechanism, we further explored the associations of iciHHV‐6 with biomarkers of inflammation. Microglial cells, the primary immune cells in the brain, release pro‐inflammatory chemokines such as IL‐6 and TNF when activated. These chemokines contribute to neuroinflammatory responses and may promote the progression of neurodegenerative diseases [44]. Cerebrospinal fluid samples were not collected in the UKB, but we tested the blood‐based protein biomarkers IL‐6, TNF, CX3CL1, EN‐RAGE, LAP TGF‐beta‐1, and VEGF‐A, which were previously identified to predict incident all‐cause, AD, or VD dementia [28, 45]. Furthermore, we tested blood cell count‐based biomarkers of inflammation, of which higher NLR and SII have been shown to be risk factors of dementia, while a higher LMR was observed to be protective [46]. Moreover, we explored the association of iciHHV‐6 with elevated CRP levels, which were shown in a recent meta‐analysis to increase the risk for dementia by 62% [47]. However, none of these inflammation‐related biomarkers were associated with iciHHV‐6 status in the UKB, which is consistent with the absence of an association of iciHHV‐6 status with dementia outcome. Interestingly, CRP levels were even statistically significantly lower in subjects positive for iciHHV‐6, which we could further pin down to a 10% lower odds for subclinical inflammation. There was no association with acute inflammation, which was expected because this is, in most cases, caused by an acute infection. We would have expected that a constant exposure to high antibody responses to HHV‐6 antigens in subjects with iciHHV‐6 would lead to slightly increased CRP levels and higher odds for subclinical inflammation. However, the opposite direction was observed, and this has similarities with antituberculosis vaccine bacillus Calmette‐Guérin (BCG) vaccination, which has been shown to reduce systemic inflammation in the long run as well [43]. It is also unknown how BCG vaccination reduces inflammation. Maybe, inflammation‐related gene sets are being silenced by BCG vaccination via epigenetic changes, which has been documented before [44]. We can only speculate that there might be analogous epigenetic modifications in subjects with positive iciHHV‐6 status, which lead to a decreased release of pro‐inflammatory cytokines and generally lower systemic inflammation.

### 4.5 Strengths and Limitations

The main strengths of this study are the exceptionally large sample size (*n* = 247,731) and the long‐term dementia follow‐up (median of 13.6 years), which enabled us to explore the association of the rare exposure to iciHHV‐6, which occurs only in about 1% of the population, with dementia outcomes for the first time and with sufficient statistical power.

There are also some limitations we need to communicate. Participants in the UKB are healthier, engage more in a healthy lifestyle, and have a higher socio‐economic status than the general population, which can cause healthy volunteer bias [5]. While this bias can have an impact on prevalence estimates (e.g., ici‐HHV6 prevalence), it should not affect relative effect estimates (e.g. the HRs estimates for the associations of ici‐HHV6 with dementia outcomes). Furthermore, the ici‐HHV6 assay did not differentiate between the integration of the HHV‐6A or HHV‐6B DNA, which could be of biological relevance. Regarding inflammation, it is a limitation that all biomarkers were only measured once at baseline, and they will have had high fluctuation during follow‐up. This could have affected associations with the dementia outcomes, but not the cross‐sectional association of ici‐HHV6 seropositivity with subclinical inflammation. Finally, since most participants of the UKB were of white British ethnicity (92.9%), the results cannot be generalized to other populations except maybe other populations with mostly European ancestry.

## 5 Conclusion

These findings from the large UKB do not support suggestions that iciHHV‐6 is a risk factor for dementia and increased inflammation, although individuals with iciHHV‐6 had significantly higher antibody responses against the HHV‐6 antigens IE1A and IE1B, which could, in theory, cause an immune system activation and thereby increase the dementia risk. In contrast, this study showed that subjects with iciHHV‐6 even had lower odds for subclinical inflammation – an unexpected finding that deserves further investigation. Further interesting observed associations of iciHHV‐6 positivity that may deserve attention in further studies were observed with ethnicity, telomere length, education, and lifestyle factors.

## Author Contributions

Q.Z. and B.S. contributed to the conceptualization and methodology of the study. Q.Z. performed the formal analysis, investigation, and wrote the original draft of the article. Q.Z., T.W., H.B., B.H., and B.S. contributed to data curation. Q.Z., T.W., M.M., R.X., and H.B. contributed to writing–review and editing of the article. All authors reviewed and approved the final version of the article.

## Ethics Statement

The North West Multicentre Research Ethics Committee (REC reference11/NW/03820) provided ethical approval to UK Biobank. UK Biobank is conducted in accordance with the 1964 Helsinki declaration and its later amendments.

## Conflicts of Interest

The authors have no conflicts of interest. The funders had no role in the design of study; in the collection, analyzes, or in interpretation of data; in the writing of the article or in the decision to publish the results.

## Supporting information


Supporting File 1

## Data Availability

The data that support the findings of this study are available from UK Biobank. Restrictions apply to the availability of these data, which were used under license for this study. Data are available from the author(s) with the permission of UK Biobank. Data from the UK Biobank are available to bonafide researchers upon application through the UK Biobank Access Management System (https://www.ukbiobank.ac.uk/enable-your-research/apply-for-access).
