# Supplementary material for: Associations of Inherited Chromosomally‐Integrated Human Herpesvirus 6 With Dementia Incidence, Inflammation, and Other Dementia Risk Factors in the UK Biobank
Source: J Med Virol. 2026 Jul 30;98(8):e71085. doi: 10.1002/jmv.71085 (PMC13424958; doi:10.1002/jmv.71085)
Supplement: Supplementary file 1 — Supporting File 1 [file JMV-98-e71085-s001.docx]

**Supplemental Material to Article**

“Associations of inherited chromosomally-integrated human herpesvirus 6 with dementia incidence, inflammation and other dementia risk factors in the UK Biobank”

Table of Contents

[Supplemental Text 1. Extended information on covariates 2](#_Toc212203462)

[Supplemental Table 1. Baseline characteristics and their associations with iciHHV-6 status restricted to study participants with European British ethnicity 3](#_Toc212203463)

[Supplemental Table 2. Association of iciHHV-6 status with HHV-6 seropositivity 5](#_Toc212203464)

# **Supplemental Text 1.** Extended information on covariates

Socio-economic dimensions embraced age, sex, ethnicity, years of education, and average total household income before tax. Lifestyle factors were further assessed through inquiries into duration of moderate activity, smoking habits, alcohol consumption patterns, and BMI. This information was obtained in the touchscreen questionnaire. Information on current diseases, including history of depression, anxiety, coronary heart disease (CHD), stroke, hypertension, diabetes, arthritis, and thyroid dysfunction was obtained in the verbal interview. Grip strength was measured in the left or right arm, using the stronger arm for analysis. Hearing and sleep problems were collected through the touchscreen questionnaire via the questions “Do you have any difficulty with your hearing?” and “Do you have trouble falling asleep at night or do you wake up in the middle of the night?”. APOE ε4 allele status was determined based on the allelic configuration of the single nucleotide polymorphisms (SNPs) rs7412 and rs429358. Participants were classified into the APOE ε4 allele status ε2ε4, ε3ε4, ε4ε4, or non-APOE ε4 allele carrier. Using the multiplex qPCR methodology Leukocyte telomere length (LTL) was measured as the ratio of telomere repeat copy number (T) relative to that of a single copy gene (S, Hgb). The relative T/S ratio was log-transformed and subsequently Z-standardized. Full details of the LTL measurements and adjustments can be found in Codd, V. et al (<https://doi.org/10.1101/2021.03.18.21253457>).

# **Supplemental Table 1.** Baseline characteristics and their associations with iciHHV-6 status restricted to study participants with European British ethnicity

| **Risk factors** | | **Total population**  **(n=230,103)** | **iciHHV-6 status** | | |
| --- | --- | --- | --- | --- | --- |
|  | |  | **iciHHV-6  positive (n, %) (n=3,127)** | **Odds ratio ^a^** | **P value ^a^** |
| **SOCIODEMOGRAPHIC FACTORS** | |  |  |  |  |
| **Age (years)** | |  |  |  |  |
| < 60 | | 84,880 (36.9%) | 1,185 (1.4%) | Ref. | Ref. |
| ≥ 60 | | 145,223 (63.1%) | 1,942 (1.3%) | 0.95 (0.88; 1.02) | 0.210 |
| **Sex, n (%** | |  |  |  |  |
| Female | | 122,117 (53.1%) | 1,648 (1.3%) | Ref. | Ref. |
| Male | | 107,986 (46.9%) | 1,479 (1.4%) | 1.04 (0.97; 1.11) | 0.296 |
| **Education (years), n (%)** | |  |  |  |  |
| < 10 | | 70,754 (30.7%) | 1,028 (1.5%) | Ref. | Ref. |
| ≥ 10 | | 159,349 (69.3%) | 2,099 (1.3%) | 0.88 (0.81; 0.95) | **0.001** |
| **Annual household income (£), n (%)** | |  |  |  |  |
| < 18,000 | | 70,028 (30.4%) | 979 (1.4%) | Ref. | Ref. |
| 18,000- < 51,999 | | 121,892 (53.0%) | 1,616 (1.3%) | 0.99 (0.91; 1.08) | 0.881 |
| ≥ 52,000 | | 38,183 (16.6%) | 532 (1.4%) | 0.99 (0.89; 1.11) | 0.917 |
| **GENETIC FACTORS** | |  |  |  |  |
| **APOE ε4 allele status, n (%)** | |  |  |  |  |
| No carriers | | 165,101 (71.8%) | 2,241 (1.4%) | Ref. | Ref. |
| ε2/ε4 | | 5343 (2.3%) | 63 (1.2%) | 0.87 (0.68; 1.12) | 0.286 |
| ε3/ε4 | | 54,635 (23.7%) | 749 (1.4%) | 0.99 (0.91; 1.10) | 0.995 |
| ε4/ε4 | | 5,024 (2.2%) | 74 (1.5%) | 1.10 (0.87; 1.38) | 0.423 |
| **Telomere length (log Z adjusted)** | |  |  |  |  |
| < -0.41 | | 89,165 (38.8%) | 1,172 (1.3%) | Ref. | Ref. |
| -0.41- ≤ 0.41 | | 76,981 (33.5%) | 1,046 (1.4%) | 1.02 (0.94; 1.10) | 0.655 |
| > 0.41 | | 63,957 (27.7%) | 909 (1.4%) | 1.10 (1.01; 1.20) | **0.021** |
| **LIFESTYLE FACTORS** | |  |  |  |  |
| **Physical activity (hours/day), n (%)** | |  |  |  |  |
| < 1 | | 119,193 (51.8%) | 1,591 (1.3%) | Ref. | Ref. |
| 1- ≤ 2 | | 83,894 (36.5%) | 1,154 (1.4%) | 1.03 (0.95; 1.12) | 0.458 |
| > 2 | | 27,016 (11.7%) | 382 (1.4%) | 1.04 (0.92; 1.17) | 0.568 |
| **Alcohol consumption (g/d), n (%)** | |  |  |  |  |
| Abstainer | | 68,548 (29.8%) | 889 (1.3%) | Ref. | Ref. |
| Women 0 - < 20 / men 0 - < 40 | | 94,168 (40.9%) | 1,260 (1.3%) | 1.04 (0.95; 1.14) | 0.384 |
| Women 20 - < 40 / men 40 - < 60 | | 39,389 (17.1%) | 570 (1.4%) | 1.13 (1.02; 1.26) | **0.022** |
| Women ≥ 40/ men ≥ 60 | | 27,998 (12.2%) | 408 (1.5%) | 1.14 (1.01; 1.29) | **0.030** |
| **Smoking status, n (%)** | |  |  |  |  |
| Never | | 118,737 (51.6%) | 1,564 (1.3%) | Ref. | Ref. |
| Former | | 91,083 (39.6%) | 1,252 (1.4%) | 1.04 (0.96; 1.12) | 0.318 |
| Current | | 20,283 (8.8%) | 311 (1.5%) | 1.15 (1.01; 1.30) | **0.027** |
| **BMI (kg/m^2^), n (%)** | |  |  |  |  |
| < 20 | | 4,379 (1.9%) | 55 (1.3%) | Ref. | Ref. |
| 20 - < 25 | | 65,802 (28.6%) | 872 (1.3%) | 1.05 (0.81; 1.40) | 0.714 |
| 25 - < 30 | | 101,942 (44.3%) | 1,436 (1.4%) | 1.11 (0.86; 1.48) | 0.438 |
| 30 - < 35 | | 42,239 (18.4%) | 566 (1.3%) | 1.05 (0.80; 1.41) | 0.721 |
| ≥ 35 | | 15,741 (6.8%) | 198 (1.3%) | 0.98 (0.73; 1.34) | 0.913 |
| **COMORBIDITY** |  |  |  |  |  |
| **Hypertension, n (%)** | |  |  |  |  |
| No | | 152,489 (66.3%) | 2,082 (1.4%) | Ref. | Ref. |
| Untreated hypertension | | 17,739 (7.7%) | 233 (1.3%) | 0.96 (0.83; 1.09) | 0.518 |
| Treated hypertension | | 59,875 (26.0%) | 812 (1.4%) | 0.99 (0.91; 1.07) | 0.813 |
| **Diabetes, n (%)** | |  |  |  |  |
| No | | 216,099 (93.9%) | 2,946 (1.4%) | Ref. | Ref. |
| Yes | | 14,004 (6.1%) | 181 (1.3%) | 0.94 (0.80; 1.09) | 0.445 |
| **Coronary heart disease, n (%)** | |  |  |  |  |
| No | | 214,956 (93.4%) | 2,919 (1.4%) | Ref. | Ref. |
| Yes | | 15,147 (6.6%) | 208 (1.4%) | 1.01 (0.86; 1.15) | 0.984 |
| **History of stroke, n (%)** | |  |  |  |  |
| No | | 297,932 (98.2%) | 3,070 (1.0%) | Ref. | Ref. |
| Yes | | 5,356 (1.8%) | 57 (1.1%) | 1.02 (0.77; 1.31) | 0.892 |
| **Lifetime history of depression, n (%)** | |  |  |  |  |
| No | | 206,241 (89.6%) | 2,805 (1.4%) | Ref. | Ref. |
| Yes | | 23,862 (10.4%) | 322 (1.3%) | 0.99 (0.88; 1.11) | 0.904 |
| **Anxiety, n (%)** | |  |  |  |  |
| No | | 227,188 (98.7%) | 3,083 (1.4%) | Ref. | Ref. |
| Yes | | 2,995 (1.3%) | 44 (1.5%) | 1.08 (0.79; 1.44) | 0.621 |
| **Arthritis, n (%)** | |  |  |  |  |
| No | | 197,304 (85.7%) | 2,909 (1.5%) | Ref. | Ref. |
| Yes | | 32,799 (14.3%) | 479 (1.5%) | 1.01 (0.90; 1.11) | 0.960 |
| **PHYSICAL FUNCTION** | |  |  |  |  |
| **Grip strength, n (%)** | |  |  |  |  |
| Low | | 117,813 (51.2%) | 1,551 (1.3%) | Ref. | Ref. |
| Average/high | | 112,290 (48.8%) | 1,576 (1.4%) | 1.07 (0.99; 1.15) | 0.076 |
| **Hearing impairment, n (%)** | |  |  |  |  |
| No | | 160,303 (69.7%) | 2,138 (1.3%) | Ref. | Ref. |
| Yes | | 69,800 (30.3%) | 989 (1.4%) | 1.06 (0.98; 1.15) | 0.131 |

Bold print: Statistically significant (P <0.05).

Abbreviation: APOE, apolipoprotein E; BMI, body mass index; iciHHV-6, inherited chromosomally-integrated human herpesvirus 6.

^a^ Logistic regression adjusted for age, sex, ethnicity, education, household income, APOE ε4 allele status and telomere length.

# Supplemental Table 2. Association of iciHHV-6 status with HHV-6 seropositivity

|  | **HHV-6 seropositivity^a^** | |  | **Total population** |
| --- | --- | --- | --- | --- |
|  | **HHV-6 negative** | **HHV-6 positive** |  |  |
| **iciHHV-6 negative** | 438 (99.5%) | 4,269 (98.7%) |  | 4,707 (98.8%) |
| **iciHHV-6 positive** | 2 (0.5%) | 57 (1.3%) |  | 59 (1.2%) |
| **Total** | 440 (100%) | 4,326 (100%) |  | 4,766 (100%) |

Abbreviation: HHV-6, human herpesviruses 6; iciHHV-6, inherited chromosomally-integrated human herpesvirus 6.

^a^ For the comparison of the HHV-6 serostatus in the iciHHV-6 positive and negative groups the unadjusted odds ratio (95%CI) is 2.92 (0.71 – 12.02) and the P value of a χ2 test is 0.119.
